# Evaluating Patient and Public Involvement and Engagement Activity Within the 3DPiPPIn Trial: A Qualitative Exploration of Contributors' Perspectives on Their Impact

**DOI:** 10.1111/hex.70674

**Published:** 2026-04-20

**Authors:** Ime Umoabasi, Stephanie K. Mansell, Swapna Mandal, Cherry Kilbride, Stephen T. Hilton, Eleanor Main, Silvia Schievano, Tamsin Callaghan

**Affiliations:** ^1^ University College London London UK; ^2^ Royal Free London NHS Foundation Trust Royal Free Hospital London UK; ^3^ Brunel University of London Uxbridge Middlesex UK

**Keywords:** impact evaluation, interviews, Patient Advisory Group, Patient and Public Involvement and Engagement (PPIE), qualitative research, Thematic Analysis

## Abstract

**Introduction:**

Patient and Public Involvement and Engagement (PPIE) is now considered essential to the delivery of high‐quality, patient‐centred and translational research. However, despite widespread recognition of this, PPIE remains poorly understood, inconsistently utilised and inadequately reported.

**Objective:**

This study aimed to report, discuss and analyse the PPIE activities undertaken within 3DPiPPIn—a randomised control trial investigating the feasibility of using 3D printing to develop customised masks for patients receiving positive airway pressure (PAP) therapy. Emphasis was placed on analysing the wider impacts of these activities, including the impact on Patient Advisory Group (PAG) members.

**Methods:**

Data were collected from PAG members via 1:1 semi‐structured interviews, which took place either face‐to‐face or online as per members' preference. Interviews were recorded, transcribed verbatim and analysed in NVivo using Braun and Clarke's Six‐Phase Reflexive Thematic Analysis.

**Results:**

Three PAG members were interviewed; two were conducted remotely via Microsoft Teams, while the third took place face‐to‐face. Analysis resulted in the identification of three themes: ‘Disparate perceptions of PPIE influence in research’, ‘Empowered and enriched through PPIE’ and ‘Navigating the evolving experience and hurdles of PPIE’. PAG members described their influence on the trial as variable, feeling their involvement was impactful in some instances and insignificant in others. Despite this, they unanimously agreed that PAG involvement had a positive personal impact and that their experiences of PPIE were diverse and dynamic. Within the subtheme ‘Supports and stumbling blocks for PPIE’, members reflected on facilitators and barriers to PPIE. For example, the relaxed environment created by the Principal Investigator was seen to have promoted open discussion, while personal challenges sometimes diverted their focus from their role as a PAG member.

**Conclusion:**

This Reflexive Thematic Analysis explored the impact of PPIE on the 3DPiPPIn trial from the perspective of its PAG members. It exemplifies PPIE best practice and highlights areas for improvement to other researchers, advocating for meaningful rather than tokenistic PPIE. By encouraging excellence in PPIE, this report could enhance public engagement in research and, by demonstrating the impact and importance of quality PPIE, could inspire funders to ensure the provision of adequate PPIE resources.

**Trial Registration:**

Embedded within the 3DPiPPIn trial (ISRCTN 74082423).

## Patient or Public Contribution

Members of the 3DPiPPIn PAG co‐designed this PPIE impact evaluation; however, their involvement extended throughout the trial. They also provided insights that shaped the recruitment strategy, methodology, lay summary and dissemination plan for the 3DPiPPIn study.

## 1 Introduction

Patient and Public Involvement and Engagement (PPIE) is defined as research which is ‘carried out “with” or “by” members of the public rather than “to”, “about” or “for” them’ [1]. Since the importance of involving the public in research was recognised in England in the 1990s [2], attitudes towards PPIE have shifted. Where research priorities and decisions pertaining to the conduct of research were traditionally determined solely by researchers [3], insights from the populations of interest are now being acknowledged for their benefits to the relevance, quality and impact of research [4, 5]. While some researchers may themselves belong to the populations being studied, and therefore have relevant lived experience in addition to extensive theoretical knowledge, individual perspectives cannot fully represent the diversity and complexity of others' experiences. As such, researchers may not always be capable of understanding the full extent of patients' perspectives without practising PPIE [6].

Numerous studies have described PPIE as being essential to delivering high‐quality, patient‐centred research that meets the needs of those it intends to benefit [7, 8]. This is due to PPIE being credited with enhancing the conduct of research [9, 10, 11], improving participants' experience of research through due consideration of practical arrangements [12, 13, 14, 15] and facilitating dissemination of results to appropriate audiences in appropriate formats [16]. Despite this, and the fact that PPIE is now required or expected by many funding bodies [17], its conceptual foundations remain inconsistently defined [18], and its implementation and reporting remain variable and suboptimal [19, 20]. This disconnect may be attributed to a range of barriers that hinder the meaningful implementation of PPIE, including the following:

- The challenge of recruiting an appropriate PPIE group that is suitably knowledgeable, representative and diverse in ‘perspectives, experiences, expectations and interests’ [21].
- The time‐consuming nature of PPIE [22], leading to ‘involvement fatigue’ [21] (whereby contributors' capacity to engage as fully as intended becomes diminished) often due to competing external commitments or a loss of personal interest.
- Researchers perceived lack of time and capacity to accommodate the additional effort required for PPIE within already constrained project timelines [23].
- The logistical challenge of meeting the unique needs of each PPIE contributor [24].
- Researchers failing to create an equal and collaborative environment, leading to power imbalances whereby contributors perceive their role and status as being secondary to the researchers [21].
- Self‐doubt and uncertainty about the ‘real’ impact of contributors' involvement, creating reluctance to engage [22, 23].
- The disparity between contributors' and researchers' expectations of involvement, with such unmet expectations hindering engagement [22].
- A lack of adequate funding and resources [21, 22].

As PPIE has become more prominent in research, so too has the focus on evaluating its impact and effectiveness [25]. International literature in the field has more than tripled in recent years [26] and has highlighted that there is considerable room for improvement in how PPIE is both conducted and evaluated [19]. Criticisms often point to instances where PPIE has been implemented tokenistically rather than meaningfully [27], and where evaluations of its impact have failed to fully demonstrate its value and influence on research [19]. In the context of obstructive sleep apnoea and positive airway pressure (PAP) therapy, research into patient perspectives has largely taken the form of qualitative studies investigating lived experience, barriers and facilitators of treatment, and patient‐partner experiences [28, 29, 30]. While these studies provide valuable insights, they do not constitute formal PPIE and, unsurprisingly given the broader criticisms of PPIE in research, reports of ‘true’ PPIE practice and evaluations of their impact remain limited within this field. This highlights a paucity of research in this area.

This study, embedded within the 3DPiPPIn trial, evaluated the impact of PPIE within the trial through the experiences and reflections of its contributors. It is one component of a three‐part PPIE impact assessment, accompanied by (1) a PPIE impact log (unpublished), which systematically documented PPIE stakeholders, activities, surrounding discussions and the resulting impacts, and (2) a self‐assessment against the UK Standards for Public Involvement [31] (companion paper in peer review), which aimed to identify areas of good practice, areas for improvement and recommendations for future research and PPIE policy.

The 3DPiPPIn study was a randomised control trial (RCT) aiming to assess the clinical effectiveness of using 3D printing technology to customise masks for patients receiving PAP therapy [32]. A Patient Advisory Group (PAG), whose role was to advise on the development and coordination of the trial and act as a ‘critical friend’, was formed in June 2022 at the commencement of the research programme of work. Recruitment occurred through the Thoracic Medicine department's consent‐to‐contact database and advertising to the Royal Free Charity (RFC) volunteers, resulting in four members who remained consistent throughout the trial. The PAG was funded through a National Institute for Health and Care Research (NIHR) doctoral fellowship award, and all activities were costed in accordance with NIHR guidance at the time [1], allowing members to be offered reimbursement for their contributions.

PAG responsibilities were defined as follows:

- Providing views on the plans for design, implementation and evaluation of applied interventions.
- Providing advice and guidance as appropriate to progress the work of 3DPiPPIn.
- Developing appropriate documents and policies to support the development of 3DPiPPIn.
- Providing information and advice based on experience and that of others.
- Guiding and advising on the dissemination of individual research projects within the project.
- Helping guide the development of recommendations for further work.
- Requesting members to speak at meetings as appropriate and reasonable, and requesting feedback on the recommendations.
- Clarifying where a matter shall remain confidential and not for discussion outside the group.

**Figure 1:**
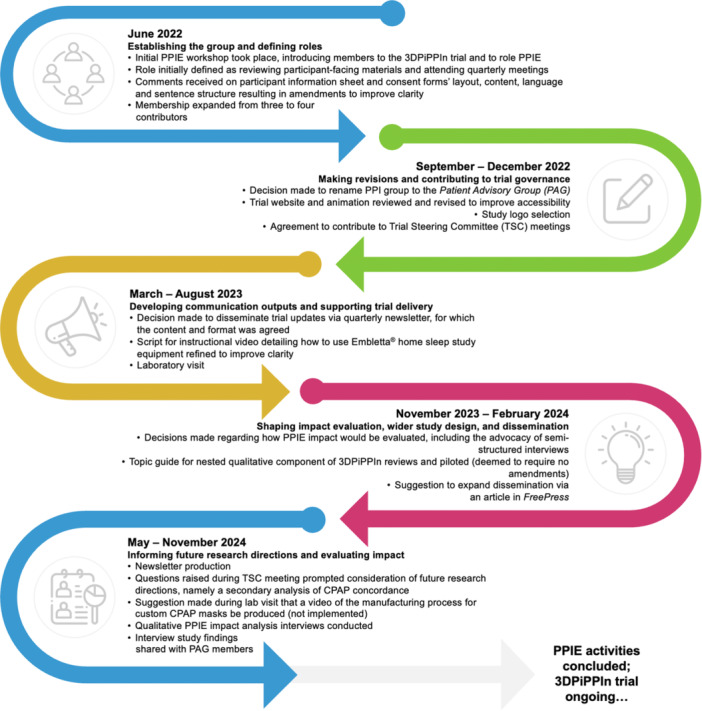
Timeline illustrating notable PPIE activities undertaken by the PAG throughout the 3DPiPPIn trial.

PAG members contributed to a range of activities aligned with these responsibilities (Figure 1).

### 1.1 Aims

The qualitative research design of this study aimed to provide an in‐depth analysis of PAG members' experiences of involvement in the 3DPiPPIn trial in as PPIE contributors, and their perceptions of its impact on themselves, the trial and beyond.

Within this, the objectives were to do the following:

1. Identify the impact of PPIE within the 3DPiPPIn trial on individual PAG members, and discuss any additional impacts that were experienced or reported as absent.
2. Identify potential enablers, challenges and barriers to ensuring meaningful PPIE from the perspective of PAG members.
3. Identify areas of good practice as well as solutions to any problems identified by PAG members.

## 2 Methodology

To fulfil the study aims and objectives, individual interviews were conducted and analysed using Reflexive Thematic Analysis (TA). In doing so, PAG members' perspectives were explored through the researcher's interpretive engagement with the interview transcripts and the construction, rather than the assumed emergence, of themes from the data. The study is informed by a constructivist ontology, which views reality as socially produced through interaction [33], and an interpretivist epistemology, which assumes that knowledge is co‑created between the researcher and participants and shaped by individual experiences [33]. These assumptions align with the reflexive nature of TA and support an analytic process grounded in meaning‑making rather than objective extraction [34].

The researcher (I.U.) was relatively new to qualitative research and did not hold strong prior beliefs regarding PPIE in research; however, reflexivity was maintained throughout the study. This was achieved through regular discussions with the study's Principal Investigator (PI) (S.K.M.) and co‐researchers (C.K., T.C.), which challenged assumptions and interpretations as they arose, and prompted ongoing reflection on positionality. These practices helped ensure that theme development remained grounded in participants' accounts rather than personal predispositions.

## 3 Methods

### 3.1 Members and Recruitment

Members of the 3DPiPPIn PAG were invited to contribute to this PPIE activity. The 3DPiPPIn PI (S.K.M.) acted as a gatekeeper, facilitating the introduction of a researcher (I.U) not known to the PAG members via email. In August 2024, following this introduction, all four PAG members were provided with relevant information (in the form of a participant information leaflet) and invited, via email, to participate in a 1:1 semi‐structured interview at their convenience. A follow‐up telephone call was made to discuss interest further and make practical arrangements.

For the purposes of this study, PAG members were considered research participants only in the context of the qualitative interviews. Beyond this, their role within 3DPiPPIn remained that of PPIE contributors.

#### 3.1.1 Inclusion Criteria

- Member of the 3DPiPPIn PAG
- Able to provide informed consent

#### 3.1.2 Exclusion Criteria

- Not a member of the 3DPiPPIn PAG
- Unable to provide informed consent

### 3.2 Data Collection Methods

Semi‐structured 1:1 interviews took place in September 2024, one face‐to‐face and two via the video conferencing platform Microsoft (MS) Teams. The semi‐structured interview technique was selected because it is valued for its ability to enable deep, flexible, conversational exchanges between interviewers and interviewees and facilitates the collection of an increased depth of data [35].

Prior to the interviews, written informed consent was obtained from members via a consent form signed and returned either electronically or in‐person. As was the case for the face‐to‐face interview, interviews conducted via MS Teams were held in a private, quiet room to maintain confidentiality and minimise interruptions. The face‐to‐face interview was audio recorded using a Dictaphone (Olympus Digital Voice Recorder DM6‐70), and the remote interviews were audio‐visual recorded using MS Teams' record and transcribe feature for meetings.

The interview structure followed a topic guide (online supplement) which was developed based on previously published research [36, 37, 38, 39, 40, 41] and researcher experience. Upon completion of a pilot interview with an experienced qualitative researcher (C.K.), the topic guide was reviewed, and minor amendments were made to ensure clarity and elicit informative answers. Amendments included reordering interview questions to better reflect the natural flow of conversation, rewording leading or assumptive questions (e.g., ‘What skills have you gained?’ to ‘Have you gained any skills?’), and simplifying language to make key concepts easier to understand (e.g., ‘areas of good practice’ into more accessible terms). PAG member insight was not sought during the topic guide development to avoid reducing the already small sample available for interview through exposure to the questions, which could have influenced responses. The topic guide allowed the researcher to explore relevant topics in a focused, comprehensive and systematic manner, enabling effective use of interview time [42]. It also allowed the interviewees to shape the discussion and consider topics adjacent to the questions posed. Questions within the topic guide included the following:

How has being a member of the PAG impacted you personally?
Have there been any challenges or difficulties you have faced as a PAG member?
How do you think researchers perceive the value of PAG contributions?
Do you have any suggestions for improving PPIE in future research projects?

### 3.3 Data Analysis

All interviews were transcribed verbatim (Type Out Transcription Services, UK) and checked for accuracy against the recordings by the interviewer. Interview transcripts were returned to members via email for member checking and validation; however, no responses were received. No further attempts were made to encourage engagement with the member checking process due to practical constraints imposed by the study timeline, and the absence of responses was taken to indicate that the transcripts were accurate. The finalised transcripts were imported into NVivo (V.14.24.0, Lumivero, USA) for analysis using Braun and Clarke's six‐phase Reflexive TA approach [43].

In the first phase, familiarising oneself with the data, the researcher read through the transcripts several times and made preliminary notes on any initial ideas. During phase two, the researcher systematically organised the data into meaningful groups and generated 26 initial codes. These codes were classified into topic clusters according to the research questions they addressed, using a methodologically rigorous approach that was scientifically descriptive, ‘valid, and representative of the data’ [44]. This phase was repeated by a second researcher (S.K.M.), who identified an additional 31 codes. Where conflicts in coding arose, they were resolved through discussion between the two researchers (I.U. and S.K.M.), and all codes were reclassified to integrate the new insights. Phase three involved grouping related codes to form broader, overarching themes, with an early‐stage thematic map helping to illustrate the relationships between them. In the fourth phase, candidate themes were reviewed to ensure the coded data extracts within each theme formed a cohesive narrative and that the themes accurately represented the entire dataset. During phase five, themes were defined and named, in preparation for the final report, which was reported in line with the Standards for Reporting Qualitative Research (SRQR) [45].

### 3.4 Data Management

All data were handled in accordance with the Data Protection Act (2018) [46] and UK General Data Protection Regulation (2018) [47]. Data were stored on secure Royal Free London NHS Foundation Trust and University College London systems and were anonymised, password‐protected and only accessible by the research team.

### 3.5 Ethics

The 3DPiPPIn study (for which the PAG group was established) was reviewed by the Hampshire B Research Ethics Committee (REC reference: 22/SC/0405), who granted a favourable ethical opinion. The evaluation of PPIE impact reported in this manuscript was included within this approval, as it was designed to be a qualitative study nested within the 3DPiPPIn trial and formed part of the trial's PPIE cycle.

## 4 Results

One PAG member declined to participate, so a total of three members were interviewed. The duration of interviews was between 32 and 40 min. Members were all males, who were either previous or current PAP therapy users. They were aged 37, 72 and 78 years old and were from diverse ethnic backgrounds, identifying as Asian British, White Other and Black British. PAG members' employment statuses also varied: one was working, one was semi‐retired and one was retired.

**Figure 2:**
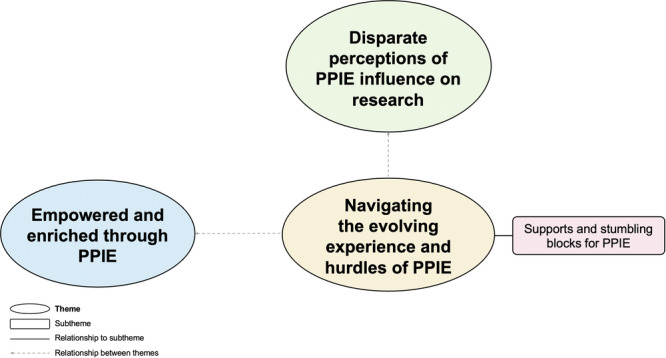
Finalised thematic map illustrating three key themes identified from PAG member interviews.

Reflexive TA resulted in the development of three themes (Figure 2): ‘Disparate perceptions of PPIE influence in research’, ‘Empowered and enriched through PPI’ and ‘Navigating the evolving experience and hurdles of PPIE’. The themes identified are detailed below and supported with representative quotes.

### 4.1 Theme 1: Disparate Perceptions of PPIE Influence on Research

Members reflected on the impact of their involvement in the 3DPiPPIn trial and described their input to have been meaningful to the research. They discussed attending quarterly meetings and providing insights on research design and conduct, including patient‐facing multimedia and materials (namely participant information leaflets, consent forms, newsletters, videos and websites), which led to tangible changes. As recorded in the PPIE impact log, the content, language, sentence structure and layout of participant information leaflets and consent forms were amended on account of PAG feedback. Similarly, the trial animation was made more accessible for prospective participants through changes to the music, script and voiceover. Trial updates were disseminated beyond the website and newsletter, as originally intended by the PI, via an article in *Freepress* (Royal Free London NHS Foundation Trust's magazine for staff, members and governors). Through this involvement, members reported they were given the opportunity to voice their opinions and reflected that some of their recommendations were implemented. They further explained that the implementation of their recommendations, as described above, combined with clear communication from the PI, validated their perceptions of the significance of their contributions to the research.

…just even sitting in the meetings, and I'm sure [the principal investigator's] picked up stuff and things have changed, ideas have come across.

[Participant 2]

[The principal investigator] is just very good at explaining what [PPIE contributions were] for and what part of the process it's for. So, you understand what's happening and sort of what value is being added at the time.

[Participant 3]

Members similarly reflected on the wider acknowledgement of their involvement. Some members had been recruited through the RFC, and discussions of how their contributions were recognised beyond the 3DPiPPIn trial highlighted that they felt the value of their PPIE activity was affirmed by others. This was largely attributed to the interest expressed in trial updates by other volunteers. While this external recognition does not directly reflect impact on research, it likely contributed positively to PAG members' perceptions of their influence.

**Interviewer**

Yes, and is there anything kind of specific that made you feel that your contributions were really appreciated and valued?

**Interviewee**

Because they [RFC volunteers] always asked have you put this in the timesheet or how far has the project gone? So other people are also, especially from my volunteers team, they're asking for it [updates on PPIE activities and outcomes of the trial]. So I think that that shows that there is something that they're interested in as well.

[Participant 1]

In conflicting narratives, all members expressed that PAG involvement within the 3DPiPPIn trial may have only been minimal. Despite describing the vast PPIE efforts undertaken at length during the interviews, mixed perceptions were evident, as they each also shared doubts about their influence on the research as PAG members. One member explicitly described their impact on the trial as limited, and the others alluded to similar perceptions. When asked about their influence on the trial, members sometimes framed their responses in terms of their level of involvement, often stating that they ‘didn't think they were that involved’, suggesting that limited involvement was conflated with, and shaped their perceptions of, their impact.

I don't think I've had much impact on the study itself.

[Participant 2]

A similar juxtaposition in perceptions of the wider impacts of PAG involvement within members was identified. PAG members maintained that their impact had not been significant and expressed that the scope of their impact was also limited, again equating this with their limited involvement.

**Interviewer**

…and then through your involvement in this Patient Advisory Group then, have you noticed any changes in the way researchers might work or think?

**Interviewee**

[…] No, I've not. I mean, [the principal investigator] would speak to me during the meeting saying this is what we do, this is how the things are going to happen but apart from that I wouldn't say that I sort of help research or have impacted a great deal or something like that.

[Participant 1]

I'm not quite sure whether I have been able to impact anything else surrounding other people.

[Participant 1]

It's maybe hard for me to say and judge that [whether they influenced researchers’ behaviour, thinking, or ways of working] because I don't know if I'm involved that deeply, if that makes sense?

[Participant 3]

This theme captured PAG members' reflections on the influence of their involvement, which ranged from a strong sense of contribution to more modest or uncertain perceptions of impact. These contrasting accounts, within individuals, suggest variability in how the PAG members experienced and interpreted their role within the research.

### 4.2 Theme 2: Empowered and Enriched Through PPIE

In contrast to the variability in perceived influence on the trial described in Theme 1, PAG members expressed homogenous perceptions of the impact of PPIE activities on themselves as individuals. They described a profound sense of satisfaction, derived less from the observable external impact of their involvement and more from finding personal value in being part of a collaborative process that validated their lived experiences. Members described their role to as having been self‐fulfilling, with some acknowledging that their input would help shape research outcomes they hoped would make tangible differences to future healthcare practices.

…the satisfaction of getting involved…

[Participant 1]

…the only more personal impact [of PPIE] is just it's good just knowing obviously I'm helping towards something possibly finding a solution to masks and finding a better product and something could help in the future.

[Participant 3]

PAG members detailed the extensive opportunities for personal growth and development that arose from PPIE activities. They described being exposed to responsibilities outside of their comfort zone and contrasted this to their professional lives. Members also shared the positive impacts of their increased understanding of sleep apnoea, Continuous Positive Airway Pressure (CPAP) therapy and the research cycle.

Getting more involved, put it that way, I'm growing, it's the first time I've been on any committees like this.

[Participant 2]

…I like to learn other things as well as well as … I don't know, just being part of stuff that's totally different to my comfort zone.

[Participant 3]

Where it's a bit different with this one whereas volunteers we're learning as we go on. Where we're, sort of, finance ones [in reference to the PAG member's work environment] it's everyone is, sort of, an expert in their field. And everyone is coming from a different point of view where some people are learning as they go on and they're not necessarily an expert in this type of … yeah, this type of research. So, yeah, different you're learning as you go on.

[Participant 3]

The diverse peer support network formed by PAG members had a considerable beneficial personal impact. Members voiced sentiments of finding a sense of belonging and validation within the PAG, a comfort some were experiencing for the first time despite decades of being diagnosed with sleep apnoea. They reported diminished feelings of isolation and burden related to their challenging lived experiences through communicating with one another, as they were able to provide advice and share perspectives both within and outside of scheduled meetings.

…there's people from all different walks of life and different environments, different cultures.

[Participant 3]

there were there are other members within the group who'd also got the same sort of issues, so in a kind of way it's […] ‐ helped me to understand that this is not just me who is having these issues.

[Participant 1]

…we quite often meet up [outside of quarterly PAG meetings] because he [a fellow PAG member] suffered quite a bit with the sleep apnoea so and, you know, he appreciates because I often have a chat with him about so many different things…

[Participant 1]

I just found it very interesting, to participate with people in different to the environment that I've come from, so you give advice or you can give input from a different environment.

[Participant 2]

This theme illustrates how involvement in the PAG led to a range of individual benefits, including emotional fulfilment, skill development and connection with others who shared similar experiences. These personal impacts were described as meaningful and sustained across members.

### 4.3 Theme 3: Navigating the Evolving Experience and Hurdles of PPIE

PAG members discussed the variety of ways in which they were involved in the 3DPiPPIn trial, illustrating the diversity of their experience as PPIE contributors. They described undertaking activities including attending quarterly meetings and laboratory visits, producing and reviewing patient‐facing multimedia and materials and piloting aspects of the trial.

I went through the consent forms, the initial consent forms […] and the initial leaflet. So, I reviewed those and did the QA [quality assurance] of those. The website, I provided suggestions of websites that [the principal investigator] could use. So, she looked at those. And, obviously, I went through the website with her when she created the website again.

[Participant 3]

…we're visiting the lab one of these days again.

[Participant 2]

I have given some input I think on the newsletter.

[Participant 1]

Members reflected that much of their PPIE activity focused on providing support and feedback on research processes. For some, the consultative nature of their role imposed practical limits, restricting their ability to provide as much input as they felt they could offer, representing an inherent hurdle in their participation as PAG members.

…it's just providing support, I think it's more providing support and just if there's any providing maybe initial feedback.

[Participant 3]

I could have given more input.

[Participant 2]

Aside from their involvement in the qualitative interviews, PAG members' role in 3DPiPPIn was not participatory in the traditional sense. One member reflected more explicitly on PPIE activity not aligning with their expectations for involvement, having hoped to be more directly involved in the trial beyond providing advisory input.

I could go on the trial [as a participant] and try the mask and give feedback on the actual trial, rather than being on the outside like I am now.

[Participant 1]

…I would have got more involved on the financial side, that's my area where I could have helped [the principal investigator] and given impact, but the authorities wouldn't allow me to get access.

[Participant 1]

These reflections suggest that preconceptions about what PPIE activity entailed may have influenced members' perceptions of their role. As their responsibilities remained within the scope of a PPIE contributor, rather than that of a 3DPiPPIn participant or trial manager, fulfilling this role may at times have felt limited relative to their expectations. This appears to reflect an experience shaped by uncertainty regarding the boundaries of their role.

Members described that their involvement and engagement in the trial had not been limited to a single point in the research cycle but rather spanned across multiple stages. They reflected that their level of involvement was fluid and had changed over time, portraying an evolving experience of PPIE featuring periods of both higher and lower participation and fluctuations in perceived contribution.

…but as I go along now, I feel I am getting more involved, and I am adding more value.

[Participant 2]

For example, while two members felt as though their involvement had been slow to start with but subsequently gained momentum, the other ‘definitely felt really involved [in the trial] especially earlier on’ **[Participant 3]** but felt involvement had since dwindled due to competing commitments:

It's probably only due to myself, more work commitments and time […] I've just really been really busy with work.

[Participant 3]

Despite wavering levels of participation, PAG members shared the intrinsic and altruistic motives that drew them to join the PAG and helped sustain their overall engagement throughout the trial. These included intellectual curiosity, personal interest in the research topic and the potential of the research to benefit their own healthcare (as well as that of others). Members also cited experience of previous research activity, with some specifically recounting prior PPIE experience, as further motivation for their involvement. Internal motives such as these may have been crucial to PAG members' enduring commitment despite the hurdles faced, particularly when their involvement was intermittent, less extensive than they might have hoped or constrained by other commitments.

I have always got an interest in helping, […] if it can help people similar with sleep apnoea and respiratory conditions, going forward, I'm always happy to help…

[Participant 1]

…they were dealing with the mask, and I thought that's very interesting. You know, I'm prepared to help because it's a field of interest.

[Participant 2]

Because, obviously, with the mask, I use the mask. And I've had issues and leaking problems with it personally myself. So, again, a situation where we can possibly find solutions getting a mask that would suit all types of people, find a better product was something I was interested in and would try and help support where possible.

[Participant 3]

I've done, in previous years […] [PPIE] work with kidney issues as well, there was a research at the [redacted] Hospital.

[Participant 1]

This theme highlighted the varied and evolving nature of PAG members' involvement in the trial. Their fluctuating levels of participation, shaped by personal circumstances and unfulfilled expectations regarding the scope of their role, reflected the dynamic reality of PPIE in practice. When faced with hurdles, members' strong intrinsic motivations and shared interest in the research topic enabled them to navigate an experience that was not without its stumbling blocks, ultimately leaving them feeling content with how PPIE had been carried out.

I just think [it] has been done really well. […]Yeah, it has just been a well‐run Public Advisory Group.

[Participant 3]

### 4.4 Subtheme: Supports and Stumbling Blocks for PPIE

Some narratives shared by PAG members highlighted aspects of PPIE practice that they described as working well. Within these narratives, several factors facilitating good PPIE were presented. Effective and efficient communication between members and the PI was highlighted as important to ensuring inclusive involvement within the PAG. A relaxed environment was observed, and PAG members recounted that this allowed them to be vulnerable in asking questions. The importance of not feeling under any pressure to be more involved or engaged than they could commit to was also of importance to members. Lastly, the flexible delivery of PPIE was seen as a facilitator.

So, there's no pressure for me to do more than I can.

[Participant 3]

Members explained that they understood the benefit of online PPIE interactions to the accessibility of PAG meetings; however, there was also a conflicting narrative of a keen desire for more face‐to‐face activity.

…well because it's over Zoom or Teams. Yeah, it's flexible.

[Participant 3]

I would like to see a bit more face‐to‐face communications with those people who are involved…

[Participant 1]

Members recognised that reimbursement was available to facilitate PAG involvement, but at an individual level, it was not necessary.

… in terms of reimbursement, I feel that I don't actually need to take anything for as a reimbursement thing because I'm a volunteer. I don't want anything back, I just want to give.

[Participant 1]

Though members initially did not describe barriers that may have posed a threat to their involvement, some were revealed after prompting and probing. The barriers identified included external, organisational‐level factors, such as a lack of flexibility in researcher thinking, likely rooted in confidence in their own expertise reducing the perceived need for PPIE input. Personal factors affecting the members, including time constraints and other commitments, were also noted as barriers to meaningful involvement.

I could have given more input or helped her or the research programme on the financial side by being involved, if they would allow you in as an outsider.

[Participant 2]

But the researcher [speaking from the perspective of researchers in general, not of their personal experience as a 3DPiPPIn PAG member] will say, ‘I know what I'm doing around the mask, I know what helps for the patient’, why should they need to talk to us [PPIE contributors]?

[Participant 2]

Just if I had a bit more time personally probably I'd probably do a bit more.

[Participant 3]

Despite members suggesting some of the trial's PPIE activity to be deserving of commendation, they were not exclusively positive when recounting their experiences. Constructive feedback arose, including the feeling that PAG involvement could have been improved by providing a clearer induction and explanation of activities, and allowing PAG members to interact directly with patients in order to support recruitment or observe more of the research activities, in particular, data collection.

I'd get to understand it much better, you know, I would spend time being introduced into it, or go through a better induction into it when I got involved.

[Participant 2]

Like it would have been nice, let's just say, […] if we could have been part of an interview when she's got patients, or sat [and] observed the process…

[Participant 2]

In summary, members identified several factors that supported effective PPIE, including clear communication, a flexible and inclusive approach and voluntary engagement without pressure. Alongside these positive reflections, members also shared constructive feedback, suggesting improvements which have been compiled and are presented in Table 1.

**Table 1:** Recommendations for future PPIE activity, including rationale for each recommendation.

Recommendation	Rationale
Be proactive in securing adequate funding for PPIE	Ensures the availability of financial support for contributors, should it be deemed necessary, as a means of removing a known barrier to PPIE and demonstrating the value of contributions
Offer reimbursement to PPIE contributors, while suggesting that those who do not require it may donate to charity rather than decline it altogether	Maintains the expectation of reimbursement and ensures it remains justifiable in future funding requests
Provide an intensive, well‐structured induction to PPIE activities. Ensure this is repeated when membership changes	Supports the early establishment of clarity in the role of contributors, preventing negative perceptions of impact resulting from misunderstanding
Tailor communication methods to align with contributors' preferences, while promoting inclusivity	Ensures that engagement is sustained and meaningful, and that rapport and trust can be built, without neglecting to consider accessibility needs
Create a low‐pressure environment that allows flexible engagement	Enables contributors to engage at their own pace, fostering sustained, stress‐free participation
Involve PPIE contributors in the development of PPIE strategies	Ensures clarity around roles, scope and preferred areas of involvement, reducing the risk of dissatisfaction arising from unfulfilled expectations
Structure meetings to balance the needs of all members	Ensures that all contributors can share insights and participate meaningfully by facilitating inclusive discussions
Regularly recap PPIE activities during meetings and through period reviews	Maintains visibility of contributions and provides tangible evidence of impact
Provide training and ongoing support for researchers undertaking PPIE activities	Ensures researchers are equipped with the distinct skills required to facilitate meaningful PPIE, strengthening both its quality and impact and that of the wider research
Demonstrate flexibility in researcher thinking to adapt based on PPIE activity, where possible	Demonstrates the respect for and the value of contributors' input and increases the relevance of research to those it aims to impact

## 5 Discussion

This study aimed to evaluate the experiences and impact of PPIE through the perspectives of its contributors. By amplifying the voices of 3DPiPPIn PAG members, it captured the nuanced and often complex realities of PPIE within the context of a clinical trial. To our knowledge, this is the first study to evaluate PPIE in the context of a PAP therapy trial. The resulting narratives contribute to the growing body of research into PPIE and the evaluation of its impact, offering both novel insights and parallels to earlier studies [3, 48, 49, 50, 51, 52]. The following discussion considers how the findings of the present study relate to existing literature, explores their implications for future practice and reflects on the wider context of meaningful PPIE in research.

Much of the literature surrounding best practice in PPIE includes guidance on providing reimbursement. It is recommended that researchers compensate patients/the public [22] as reimbursement facilitates sustained engagement and supports more equal research partnerships [53]. Within the present study, the PAG members interviewed highlighted that they felt reimbursement was not necessary. When remuneration was offered, members were adamant that it was not required, in keeping with their altruistic motivations for engaging with PAG activities and their intentions of ‘giving’ without receiving anything in return. Though this was true for 3DPiPPIn's PAG members, researchers should recognise that one member's decision may influence others, and widespread refusal of reimbursement could have implications on the success of future PPIE funding requests. Due to this, in hindsight, the research team would have encouraged reimbursement more proactively. It is also important to acknowledge that other PPIE groups may hold a different view on the necessity of reimbursement, as it is known to be an enabler of PPIE. Notably, the NIHR's payment guidance for researchers and professionals involving people in research [54] endorses compensating public contributors as good practice, while acknowledging that this depends on foresight during budgeting. In the case of 3DPiPPIn, although reimbursement was not deemed necessary by PPIE contributors, the ability to offer it was contingent on the study's financial capacity, something that other studies may lack. Therefore, while reimbursement considerations were made on a study‐specific basis and shaped by the preferences of 3DPiPPIn's PAG members, the NIHR is clear on its stance on the importance of reimbursing PPIE contributors.

Theme 1, ‘Disparate perceptions of PPIE influence on research’, highlighted variability in how PAG members understood and described their influence on research. Within all members, conflicting narratives were shared regarding the impact of PPIE efforts. In contrast, Crocker et al. [50] found that PPIE contributors believed PPIE had an entirely beneficial impact on research, yet, as in our study, they also struggled to articulate the impact of their own involvement at times. 3DPiPPIn PAG members, however, expressed more apparent feelings of ‘impact uncertainty’. Given the research team's recognition of PPIE impact through tangible changes, this suggests that the actual impact of PPIE contributions did not always align with members' perceptions. Such misalignment is particularly likely when the changes brought about by recommendations are not made explicit, or when recommendations are not implemented—although PAG members reported very few instances of the latter in the 3DPiPPIn trial. These findings highlight the importance of clear, closed‐loop communication regarding the outcomes of their input to dispel feelings of ‘impact uncertainty’. It is also important that such communication efforts clarify the role of PAG members. The closely related Theme 3 narrates both the actual limitations imposed on involvement by personal barriers and the perceived limitations of the advisory nature of PPIE, with some members having expected a more traditionally participatory role.

While it is not possible to make recommendations that would apply to all contexts, existing literature has provided a range of suggestions to support researchers in maximising and making visible the impact of PPIE. Among these are the consideration checklists for optimising the impact of PPIE synthesised by Aiyegbusi et al. [48]. Hanrahan et al. [55] proposed a set of recommendations to effectively involve contributors, and ensure inclusivity, transparency and relevance. Building on such guidance, we recommend that researchers consider providing a more intensive induction to PPIE, and recapping PPIE activities during meetings and through periodic reviews that would provide regular tangible evidence of impact. Ultimately, it is up to researchers to acknowledge the individuality of their PPIE groups and act accordingly to ensure that involvement impact is communicated so members can make an informed opinion on how their contributions resulted in change.

One finding that may have been unique to this study was the affirmation of influence experienced by PAG members through their social and professional circles. For example, RFC volunteers appeared to take a keen interest in the progress of the trial, and some members cited this as a factor that made them feel their contributions were greatly appreciated. Given that PAG members were largely recruited through advertising to RFC volunteers, it is unsurprising that they placed considerable value on the engagement of their peers. This homogeneity may also help explain why all members shared such altruistic motivations, and they found it challenging to articulate the distinct impact of their contributions on the research process. The interest shown by other volunteers likely reinforced the perception that members' contributions to 3DPiPPIn were an extension of their ongoing role within the Charity. Furthermore, these interactions indicate that PAG members voluntarily played a role in the informal dissemination of trial updates, suggesting that independently extending awareness of the trial beyond its immediate stakeholders reinforced their sense of influence within the trial.

Some members were content with the online delivery of PPIE activities and considered it an enabler of their involvement and engagement. Despite this, some believed more face‐to‐face PPIE activity would have enhanced their influence on the trial, a view reinforced by much of the literature on PPIE impact [56, 57, 58, 59, 60, 61]. This further illustrates the complexity and polarity of their perceptions and highlights that though individuals become part of a group through PPIE, they often maintain their own unique demands and preferences. Accommodating the preferences of each PPIE contributor can be challenging, as was shown to have been the case for 3DPiPPIn's PAG. Researchers should continue seeking to acknowledge the individuality of PPIE contributors and facilitate optimal commitment from all as best as possible.

It is important to acknowledge the presence of some overlap in the description of the themes developed. Themes 1 and 3 demonstrated overlap in members' discussion of the diverse PPIE activities they partook in, which provided supporting data for the development of both themes. Meanwhile, the development of both Themes 2 and 3 was supported by references to the intrinsic motivations for PPIE. Pioneers in TA, Braun and Clarke, advised that researchers ensure there is not ‘too much overlap between themes’ [43], raising the question as to whether ‘themes that overlap should truly be considered separate themes’. In response, it has been proposed that it is possible for sections of data to be included within multiple themes, and thus for them to overlap [62] as was the case in the present study. Rather than serve as a critique of this study, suggesting themes were poorly generated and refined, in this context, the overlapping themes were deemed to portray distinct narratives while demonstrating the complexity, interconnectivity and richness of the data gathered.

### 5.1 Limitations

Though it was intended that this study would provide guidance for other researchers hoping to conduct and evaluate the impact of PPIE, it is important to acknowledge that the present study was not without its limitations.

In interpreting the findings of this study, it is valuable to consider the characteristics of the sample. Due to the aims of this study, the recruitment pool was limited to the four PAG members, of whom three wished to participate. This is not uncommon for qualitative research, nor is it uncommon for PPIE. Reflexive TA has very few rules regarding sample size [43] and does not conceptualise data completeness in terms of thematic saturation [63] meaning that, though small, the sample size for this study could be deemed sufficient to address its aims and objectives. In this study, the three members enabled representation of 75% of the PAG, thereby reinforcing the view that this small absolute number was not necessarily a limitation in the conventional sense.

The seemingly routine practice of citing a small sample size among the limitations of a qualitative research study has been challenged in recent years [64]. It should no longer be considered an inherent limitation to the rigour and findings of qualitative research [65]. However, it is important to consider that the small sample size in this study may have limited the diversity of the perspectives gathered. It is possible that the perspectives shared may have been exclusive to the defined geographical location and the experiences of male PAG members. While diversity and inclusivity are key principles of PPIE, the male‐dominated composition of the 3DPiPPIn PAG corresponds with the higher prevalence of obstructive sleep apnoea in men [66]. For this reason, the overrepresentation of the male voice may be advantageous in capturing insights from the demographic most affected by the condition.

It is also important to consider that this study may be limited by the absence of key data and perspectives that could provide a fuller understanding of PPIE. Specifically, PAG members' educational background or socioeconomic conditions were not collected within this study. Collecting and analysing demographic data from those taking part in PPIE is increasingly being recognised as an important aspect of demonstrating its effectiveness [67]. This is because meaningful PPIE is not only a matter of how contributors are involved, but also who is being involved [67]. Capturing such data has the potential to inform future efforts to increase the diversity of PPIE groups, enabling researchers to strategically recruit those who would otherwise remain underrepresented [67]. In addition, by centring the voices of PAG members, this study did not aim to capture the perspectives of other stakeholders, such as researchers, involved in the trial. Including these viewpoints in future research could provide a more comprehensive account of how PPIE impact is experienced and interpreted.

Additionally, the timing of the interviews should be considered when interpreting the findings. Interviews were conducted in September 2024, after recruitment to the 3DPiPPIn trial had concluded but while the study remained in the follow‐up phase. As a result, participants reflected on their experiences of PPIE before all potential opportunities for involvement and impact had occurred. It is therefore possible that additional contributions or further impacts of PPIE may have emerged during later stages of the trial that were not captured within the timeframe of this study. Acknowledging this is important for contextualising the current findings, as participants' reflections may have differed had interviews been conducted once 3DPiPPIn had completed its full research cycle.

One further explicit limitation was the researcher's (I.U.) relative inexperience in undertaking qualitative research. Both data collection via qualitative interviews and using a reflexive TA were novel concepts to the researcher (I.U.). Efforts were made to reduce the potential effects of this on the rigour and credibility of the study's results. Efforts included: the researcher thoroughly reviewing the methodology to develop a better theoretical understanding, conducting a pilot interview with an experienced qualitative researcher (C.K.) to gain insights from an expert, developing familiarity with the data collection method to be used and regularly consulting 3DPiPPIn's PI (S.K.M.).

## 6 Conclusion

This qualitative study has uncovered an important narrative about 3DPiPPIn's PAG members' perspectives of the impact of their contributions. In sharing the findings of this study, the intention is to demonstrate what can be achieved when PPIE is adequately funded, and to present both an example of PPIE practice and a guide for pre‐empting areas for improvement in the work of other researchers. Furthermore, this study provides an example of measuring PPIE impact that other researchers could reproduce.

## Permission to Reproduce Material From Other Sources

Not applicable.

## Author Contributions

**Ime Umoabasi:** writing – original draft, data curation, writing – review and editing, formal analysis, visualization, investigation. **Stephanie K. Mansell:** conceptualization, resources, data curation, supervision, funding acquisition, project administration, writing – review and editing, methodology, formal analysis. **Swapna Mandal:** supervision; funding acquisition; writing − review and editing; methodology. **Cherry Kilbride:** supervision; funding acquisition; writing − review and editing; methodology. **Stephen T. Hilton:** supervision, funding acquisition, writing – review and editing, methodology. **Eleanor Main:** supervision; funding acquisition; writing − review and editing; methodology. **Silvia Schievano:** supervision; funding acquisition; writing − review and editing; methodology. **Tamsin Callaghan:** supervision, funding acquisition, writing – review and editing, methodology.

## Ethics Statement

This study for which the PAG group was established received ethical approval from the Hampshire B Research Ethics Committee (REC reference: 22/SC/0405).

## Consent

All participants included in the study provided informed consent to the use of their anonymised data.

## Conflicts of Interest

The authors declare no conflicts of interest.

## Supporting information

Supporting File

## Data Availability

Data supporting this study cannot be made available due to ethical reasons. Further enquires can be directed to the corresponding author.
