# Supplementary material for: Evaluating Patient and Public Involvement and Engagement Activity Within the 3DPiPPIn Trial: A Qualitative Exploration of Contributors' Perspectives on Their Impact
Source: Health Expect. 2026 Apr 20;29(2):e70674. doi: 10.1111/hex.70674 (PMC13094355; doi:10.1111/hex.70674)
Supplement: Supplementary file 1 — Supporting File [file HEX-29-e70674-s001.docx]

**3DPiPPIn Patient/Public Advisory Group Impact Analysis**

**Topic Guide for Semi-Structured Interviews**

**Welcome and Introduction**

- Briefly introduce self and purpose of interview
- Explain the confidentiality and consent process
- Outline interview structure

**Questions**

| **Participant Background**  **(Motivation and Engagement)** | 1. **Can you tell me a bit about yourself and your role in the 3DPiPPIn trial?** 2. **How did you become involved with the PAG?** 3. **Is there anything in particular that motivated you to join the PAG?** |
| --- | --- |
| **Personal Impact** | 1. **Do you feel being a member of the PAG impacted you personally, and if so, how?** 2. **Have you gained or developed any skills or knowledge through your participation in the PAG?** |
| **Motivation and Engagement** | 1. **How involved did you feel in the activities and decisions of the PAG?** |
| **Collaboration and Communication** | 1. **How would you describe the interaction between PAG members and researchers?** 2. **Would you be able to give me some examples of how PAG input has influenced the research process?** |
| **Perceived Value** | 1. **Do you think researchers value PAG contributions? How do you think they perceive the value?** 2. **Do you think you’ve noticed any changes in the way researchers work or think as a result of PAG involvement?** |
| **Trial Design and Conduct** | 1. **In what ways has the PAG influenced the design or running of the 3DPiPPIn trial?**   Can you think of any *other* specific instances where PAG input led to changes or improvements in the trial? |
| **Broader Influence** | 1. **Beyond the 3DPiPPIn trial, do you think the PAG has had any wider impacts on** the **research** community **or healthcare** practices**?**   **Is PPIE something you’ve been involved in before? If so, what has is looked like in other projects?**  Are there any examples of how PAG involvement has been shared or adopted by other projects or organisations? |
| **Facilitators of Meaningful PPIE** | 1. **What has helped your participation and engagement in the PAG feel meaningful** – as though your input has really made a difference to the trial**?**   Were there any specific factors, strategies or practices that were particularly effective? |
| **Challenges and Barriers** | 1. **Have there been any challenges, difficulties or barriers you’ve faced as a PAG member?** 2. **How were these challenges addressed, if at all?** |
| **Problem-Solving** | 1. **Can you suggest any solutions to those challenges and barriers?** 2. **During your experience of being a PAG member, did you notice anything that the research team did really well that you think others would benefit from knowing/doing?** |
| **Final Thoughts** | 1. **Is there anything else you would like to add about your experience with the PAG or the 3DPiPPIn trial?** 2. **Do you have any suggestions for improving PPIE in future research projects –** *not just this one***?** |

**Closing**

- Thank participant for their time and contributions
- Provide information on next steps and how the findings will be used
